# Biotechnological tools boost the functional diversity of DNA-based data storage systems

**DOI:** 10.1016/j.csbj.2025.02.002

**Published:** 2025-02-06

**Authors:** Xiaoyuan Xu, Wen Wang, Zhi Ping

**Affiliations:** a School of Medicine, The Chinese University of Hong Kong, Shenzhen 518172, China; b BGI Research, Beijing 100101, China; c BGI Research, Shenzhen 518083, China

**Keywords:** DNA data storage, DNA nanostructure, DNA storage system

## Abstract

DNA-based data storage has emerged as a groundbreaking solution to the growing demand for efficient, high-density, and long-term data storage. It is attracting many researchers’ attention, who are implementing functions such as random access, searching, and data operations apart from the existing capabilities, including reading and writing. We summarize the recent progress of how biotechnological tools, based on sequence specificity, encapsulation, and high-dimensional structures of DNA molecules, facilitate the implementation of various functions. The limitations of using biochemical reactions that hinder the development of more precise and efficient information storage systems are also discussed. Future advancements in molecular biology and nanotechnology are expected to improve the architecture, scalability, and efficiency of DNA storage, positioning it as a sustainable and dynamic alternative to conventional data storage systems.

## 1 Introduction

Traditional storage media have met their physical and technological limits because of the exponential growth of digital data. DNA, a highly compact and durable information carrier, has been suggested as a promising medium for next-generation storage solutions, particularly for archival purposes. DNA’s four-base nucleotide structure (A, T, C, and G) gives it natural advantages with respect to data density for encoding data. DNA’s stability, low maintenance cost, and storage capacity (approximately 455 exabytes per gram of single-stranded DNA) characteristics address the degradation and other problems that traditional storage media face, making it ideal for long-term data storage [1].

DNA storage includes two key processes: encoding data into synthesized DNA and reading it back (sequencing). Both processes are important for the scalability and accessibility of DNA storage technology. Recent advances in synthetic biology have expanded the functional diversity of DNA in data storage systems. Innovations such as enzymatic synthesis and error correction mechanisms have significantly improved the scalability and accuracy of DNA-based systems [2], [3]. With other advancements addressing challenges in read/write speed, synthesis cost, and error rates, DNA storage is likely to supplant traditional technologies over the long run. However, DNA synthesis itself and these techniques are still in their emerging stages, and accuracy, scalability, and affordability must be improved for DNA’s potential as a storage medium to be realized.

**Fig. 1:**
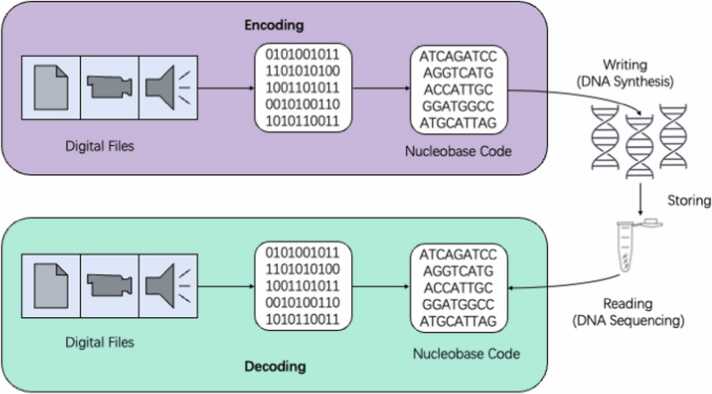
General procedure of DNA storage. Conventional DNA storage includes the following processes. (1) The binary data in digital files are converted to a DNA sequence (encoding); (2) the encoded DNA molecules are artificially synthesized (writing); (3) the DNA molecules are stored in different forms (storing); (4) the DNA molecule libraries are sequenced when information retrieval is needed (reading); and (5) the information from the sequencing data is extracted and decoded (decoding).

**Fig. 2:**
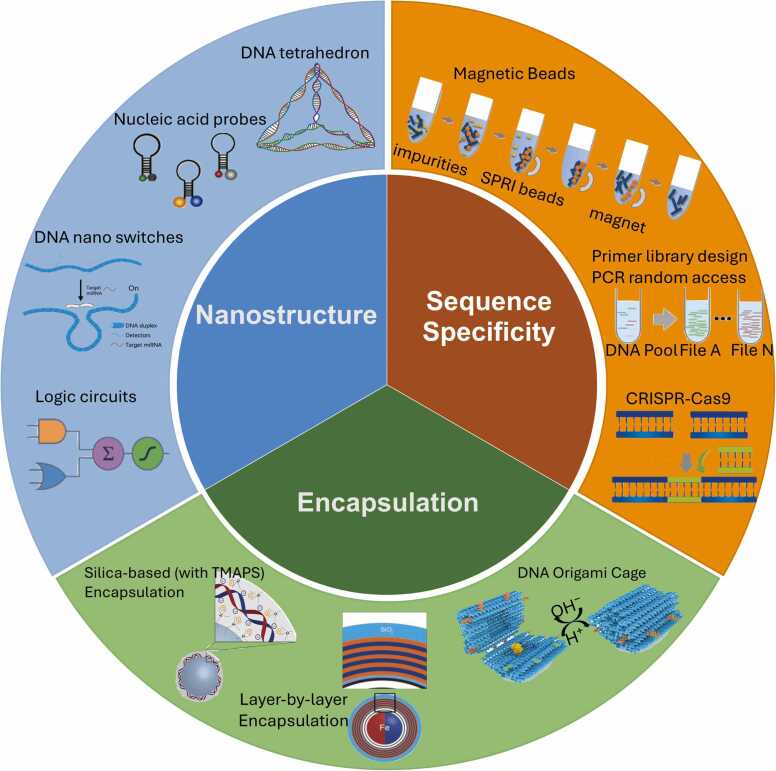
Overview of the implementation of various functions. Functions can be achieved based on 1) sequence specificity, including random access, file modification, and searching [13]; 2) encapsulation of molecules, including file management, storage architecture, and searching [14], [15], [16]; and 3) nanostructure self-assembly, including data processing and DNA computing [17], [18], [19].

**Fig. 3:**
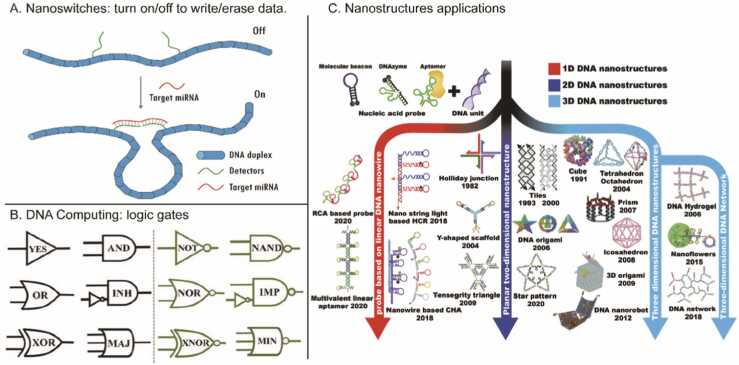
Nanostructure applications and DNA computing. a) Mechanism of nano switches based on interactions between target miRNA and DNA duplexes. The switch can be used to turn DNA on/off to write or erase data [17]. b) DNA-based logic gates as an alternative for computing processes. These can be used for data processing [41]. c) The wide range of applications for nanostructures in DNA storage and manipulation. Nanostructures are categorized into 1D, 2D, and 3D structures, with examples of each, such as DNA origami and nanoflowers, demonstrating their evolution and usage in data storage [42].

This review focuses on how synthetic biology tools, including enzymatic synthesis methods and nanostructure-based designs, are transforming the landscape of DNA data storage. Although the traditional phosphoramidite synthesis method dominates the current systems, emerging approaches, such as the kinetic competition methods, electrochemically cleavable conjugate method, and terminal deoxynucleotidyl transferase mutant methods, are potential alternatives that promise greater control and efficiency. However, their accuracy needs to be improved for them to compete with the precision of the per-base control that the phosphoramidite method allows [4], [5], [6], [7], [8]. In addition, the integration of programmable biochemical circuits offers speculative yet exciting possibilities for data integrity and on-DNA computation.

**Table 1:** Comparison of different data storage media.

**Metric**	**DNA molecules**	**Magnetic tape**	**Hard disk drive (HDD)**	**Solid-state drive (SSD)**	**Optical storage**
**Density**	∼10^19^ bits/cm^3^[9]	∼10^12^ bits/cm^3^[10]	∼10^13^ bits/cm^3^[11]	∼10^14^ bits/cm^3^[11]	100 GB/mm^3^[10]
**Lifespan**	1000 + years [9]	∼30 years [10]	∼5–10 years [11]	∼10–15 years [12]	∼10 years [10]
**Read/write speed**	Slow (∼MB/hour) [9]	Medium (∼bytes/second) [10]	Fast (∼GB/second) [11]	Very Fast (∼GB/second) [12]	Moderate (∼ MB/second) [10]
**Maintenance cost**	Low [9]	Medium [10]	High [11]	High [12]	Low [10]
**Scalability**	High (biological replication) [9]	Moderate [12]	Moderate [11]	Moderate [12]	Limited [10]

Note: MB – Megabytes, GB - Gigabytes.

In this context, we aim to provide a comprehensive overview of recent innovations while critically evaluating their potential and limitations. Unlike existing reviews, this review emphasizes how synthetic biology is broadening the application scope of DNA storage systems to move beyond simple data archiving toward enabling real-time updates, error correction, and data compression.

## 2 Functions in DNA data storage

DNA data storage is currently used for “cold” data storage of massive archival files [20]. It often includes fundamental procedures (encoding, storage, and retrieval) and additional computational operations such as random access and information searching and editing, which make use of the DNA’s nucleotide sequence, structural properties, and biochemical interactions [21], [22]. This review discusses the existing DNA storage functions based on three aspects: sequence specificity, encapsulation methods, and nanostructure applications.

## 3 Functions based on sequence specificity

DNA’s programmability and the feasibility of its de novo synthesis allow precise encoding of digital data. The resultant specificity supports advanced capabilities, such as selective retrieval and real-time data updates. Magnetic tape, which is also a medium for cold data storage, permits only sequential data access, whereas random access to targeted DNA sequences can be achieved using methods such as polymerase chain reaction (PCR) amplification, clustered regularly interspaced short palindromic repeats (CRISPR)-Cas9, and magnetic beads via designing specific motifs in the non-data regions (e.g., primers and barcoding sequences) [23], [24]. These techniques represent critical developments, as they allow capabilities to ensure data reliability and integrity when searching and modifying the data. Consequently, these features make DNA comparatively more flexible and accessible for data operation and management.

Multiplex PCR is the most widely used random access technique in the field of DNA data storage. This technique uses specific primer pairs to tag different files, allowing selective amplification of the target files from a DNA library. Since 2018, Microsoft and the University of Washington have made breakthroughs by using PCR to selectively amplify specific DNA fragments, enabling efficient and accurate data retrieval. By designing primer libraries attached to DNA sequences, they have improved retrieval speed, reduced sequencing requirements, and lowered costs. Their advancements include encoding 200 megabytes of data into DNA with error-free random access and developing a molecular controller and DNA writer chip to enhance storage and retrieval efficiency [25].

CRISPR-Cas9 has emerged as a groundbreaking tool for genetic engineering, as it allows precise editing and modification of DNA sequences. In DNA data storage, this method facilitates targeted data retrieval and modification by cutting specific regions of the DNA sequence and enabling new data insertion, which enables the encoding of new information or the correction of errors in existing DNA sequences, thus supporting real-time updates and enhanced data integrity. Its derivative methods, such as the Cas12a sensor, integrate cellular biochemical mechanisms for data storage and offer potential advantages for applications requiring long-term stability and low-cost replication. However, the application of gene editing tools remains in the exploratory phase and requires further research to address the current technical and biological limitations.

DNA mutational overwriting storage (DMOS) employs CRISPR-based base editing to overwrite the existing DNA sequences in predefined domains without requiring de novo synthesis, which reduces toxic waste and is scalable for industrial applications [23]. DMOS systems are especially promising for tasks requiring frequent updates or modifications to stored data, as they allow the reuse of blank DNA tapes, representing a cost-effective and environmentally friendly alternative to traditional methods.

Magnetic beads also play a crucial role in improving the convenience of data retrieval and operation. By using sequence-specific beads, one can quickly capture the labeled target DNA sequences from a mixture. T7 promoters have emerged as a critical tool for real-time data modification within DNA storage. These DNA sequences, recognized by an RNA polymerase enzyme, drive gene transcription and enable transcription-driven updates to the encoded data. Studies have highlighted their potential to enhance mRNA yield and quality, indicating their potential importance in biomanufacturing [26]. In DNA storage, T7 promoters facilitate rapid access and updates to specific data regions, thus improving operational speed and flexibility. The combination of transcription-based tools and DNA storage technologies holds promise for more dynamic and efficient data management systems. Researchers have also combined inorganic capsulation and sequence-specific labeling to develop a DNA data storage system using silica-coated magnetic beads, where each bead is labeled with a single-stranded DNA “barcode” for fast retrieval of specific data files [20].

The above advancements have translated into convenient searching, access, and retrieval of data in DNA in a cost-effective manner. However, in these aspects, the off-target effects and other limitations leading to crosstalk with non-specific data sequences can still be improved.

## 4 Functions based on encapsulation

Encapsulation involves coating DNA molecules with materials such as silica beads or liposomes to enhance the stability and integrity of the DNA storage medium. Encapsulation can be integrated with other biological and material science technologies, such as microfluidics and flow cytometry [21].

The current silica-based encapsulation method prevents chemical and physical degradation, especially from reactive oxygen species [27], as it forms a barrier around the DNA, which also protects it from high temperature and UV damage. However, the trade-off of such protection is increased data retrieval complexity and time consumption. Nanoparticle encapsulation, in comparison to the silica-based method, provides improvements in terms of data access and storage organization but with the sacrifice of data density, making it less ideal for high-volume archival files [28]. Simpler methods such as trehalose or sugar matrices allow room temperature storage, but the stability depends on various other environmental factors [29]. Innovations such as the DNA-of-things architecture [30], layer-by-layer encapsulation [31], and automation of the encapsulation process using microfluidic systems give new insights into possible enhancements to the architecture of DNA storage systems.

Liposomes, composed of phospholipid bilayers, effectively encapsulate molecules such as DNA in their aqueous core and impart enhanced stability, protection, and sustainability. They can effectively shield encapsulated DNA from environmental factors such as oxidation, enzymatic degradation, and light exposure [32]. This protective quality ensures the stability and prolonged bioactivity of sensitive compounds during storage and delivery. They are composed of biodegradable and biocompatible materials, such as natural or synthetic phospholipids, and this composition minimizes toxicity and environmental impact, making them an eco-friendly option for encapsulation [33]. Using microfluidic technologies to synthesize lipid-based nanoparticles has emerged as an even more effective and innovative approach for encapsulation, as it improves the control and stability of the encapsulation [34]. Hydrogel and cellulose paper exhibit high specific surface area, flexibility, and biodegradability, which reduce the cost and simplify the experimental procedure [35], [36]. These features make them strong candidates for applications requiring efficient encapsulation and functional maintenance of cells. For example, hydrogel encapsulation provides robust protection for cells while preserving their functionality. Microfluidic devices enable a precisely controlled encapsulation process, allowing for the accurate manipulation of cell quantities and encapsulation efficiency. This enables the construction of high-capacity, high-resolution in vitro tissue models, which are critical for advancing research in tissue engineering and regenerative medicine [37].

## 5 Functions based on nanostructure and DNA computing

Nanostructures can serve as a facilitator in complex DNA data operations such as error correction and data compression. DNA nanostructures, including origami and tetrahedrons, provide precise spatial organization. The high rigidity and stability of DNA tetrahedral nanostructures make them ideal for biomedical applications and data storage. Wang et al. demonstrated the use of DNA tetrahedrons to enhance biomolecule interactions and stability, suggesting possibilities for more advanced data processing applications [38].

DNA nanotechnology leverages the physical and chemical properties of DNA, rather than its genetic information, to design and construct synthetic structures. These structures have widespread applications in biomedicine and show tremendous potential in data storage and computing. For example, DNA adsorbed onto soft dendritic macromolecules has been used to construct data storage and computation engines, enabling end-to-end functionality from archival storage to non-destructive file access [39].

Recent breakthroughs in nanostructure applications further highlight their transformative potential. For example, Yan et al. introduced a new “meta-DNA” (M-DNA) strategy that enables the creation of micron-scale DNA structures that can overcome the traditional size limitations of DNA folding [40]. By employing M-DNA structures, which are 1000 times the diameter of natural DNA nanostructures, researchers have achieved self-assembly into various forms, such as tetrahedrons, polyhedral forms, and 2D/3D lattices. These structures show advanced hierarchical chain displacement reactions, thus transferring dynamic DNA characteristics to M-DNA. By altering the flexibility and interactions of individual M-DNA units, researchers have constructed a diverse range of shapes, from 1D arrays to 3D lattices.

DNA computing using DNA as the substrate for logic operations and data processing has been proven to be amenable to executing complex algorithms within the storage medium itself [43]. Nanostructures play a critical role in DNA computing, as they create controlled environments for molecular interactions [42]. The predictable and programmable nature of nanostructures ensures reliability when executing computing tasks. Nanostructure-based nucleic acid probes provide a stable platform for essential molecular interactions in DNA computing due to their functions as chemical substrates. These approaches, revolving around nanostructure and DNA computing, have the potential to revolutionize DNA storage systems as they open the possibility to process DNA-based files without external computational resources.

For example, Xiang et al. introduced a flap endonuclease 1 (FEN1)-assisted DNA logic amplifier circuit, which uses FEN1 to catalyze signal amplification in DNA logic gates [43]. The circuits include configurations such as AND-OR, OR-AND, FAN-IN, FAN-OUT, and even 4-bit square root circuits. Remarkably, this system operates with input strands at concentrations below 1 nM—over 100 times lower than those used in traditional DNA logic circuits—providing a potential novel approach for rapid and compact DNA computing.

## 6 Challenges and limitations in DNA data storage

Significant challenges remain in the use of DNA data storage, particularly in ensuring the reliability and efficiency of biochemical reactions to meet the demands of accurate and scalable file processing.

## 7 Uncertainty of biochemical reactions vs. accurate file processing demands

The uncertainty of biochemical reactions poses a significant challenge to achieving the precision required for accurate data processing in DNA storage systems. This uncertainty arises from the byproducts generated during reactions, cross-talk between processes, and undesired molecular interactions [22]. For example, the CRISPR-Cas systems that are widely used for genome editing might face problems such as off-target effects, leading to unintended modifications [24]. Similarly, PCR, a key tool for DNA amplification, tends to form undesired structures such as hairpins or mismatched duplexes, particularly when amplifying sequences with extreme GC content or repetitive regions [44]. These issues introduce variability and noise into the biochemical processes, complicating data reliability and retrieval. Overcoming these limitations is critical to advancing DNA storage technologies to meet the stringent demands of digital data processing.

Whereas silicon-based data storage achieves remarkable precision with error rates of less than 10^−14^ [28], DNA storage involves biochemical processes such as synthesis, amplification, and sequencing, which are prone to errors due to factors such as enzymatic efficiency, reaction conditions, and molecular instability. Editing errors such as insertions, deletions, and substitutions are common. In particular, indels (insertions or deletions) occur not only during synthesis but also through natural DNA replication processes. Mismatches or incomplete reactions introduce variability, making it difficult to guarantee perfect data integrity across large datasets. Although short DNA strands are preferable in DNA synthesis processes to minimize errors, minor error rates (even a single alteration) could reduce the reliability of the data [45]. The nature of DNA’s secondary structures, such as hairpins or topological pseudoknots, can worsen these errors by introducing synthesis and sequencing challenges, especially when dealing with homopolymers or sequences with extreme GC content [46], [47].

Environmental factors, such as temperature and humidity, also worsen these issues by causing DNA degradation over time, leading to mutations and strand breaks. To address these challenges, recent studies have focused on improving reaction conditions, enhancing enzyme fidelity, and standardizing protocols. For example, Nguyen et al. explored optimized synthesis protocols that reduced error rates but highlighted the need for more scalable and robust methods [3]. The uncertainty during the synthesis process still needs to be addressed, along with more advanced encapsulation techniques to prevent DNA degradation while preserving its high-density storage property.

Sequencing processes also face challenges in efficiently detecting and correcting errors. Error-correcting codes and logical and physical redundancy strategies have been developed to address this issue. For example, the designing multiple vector optimization algorithm aims to optimize address allocation in DNA data storage to reduce the chance of overlaps and errors during retrieval [47]. The Yin–Yang codec introduced by Ping et al. employs a duplex strand encoding strategy, where complementary DNA strands (sense and antisense) store data in a manner that balances error detection and correction efficiency with storage density [46]. Advanced models, such as artificial neural networks, have been applied to predict potential errors during biochemical processes [48]. These networks identify and correct error patterns, thus boosting the reliability of DNA data storage systems and offering a sophisticated approach to dealing with molecular uncertainties. High-fidelity and high-throughput sequencing platforms provide efficient and cost-effective methods for retrieving stored data while simultaneously minimizing errors and improving the processing speed [49]. Pairing these sequencing platforms with error correction algorithms can mitigate the potential accuracy issues. However, the gap between biological unpredictability and digital precision remains and requires continued research for more advanced error correction strategies and optimized sequencing techniques.

## 8 Efficiency of biochemical reactions

The issues of inefficiency, nonuniformity, and time cost in biochemical reactions appear at different stages of DNA data storage, including synthesis, amplification, operation, and sequencing, and directly affect the feasibility and accuracy of DNA storage systems.

The efficiency of biochemical reactions does not reach 100 % and is often significantly lower. It is fundamentally based on the principles of chemical equilibrium. In other words, reactions do not proceed to complete conversion of reactants to products. In solution, the reaction between enzymes and substrates is driven by random collisions resulting from their Brownian motion. The probability of these collisions is influenced by the accessibility between them, which is determined by factors such as concentration, spatial distribution, and potential molecular obstacles, including other molecules, ions, and macromolecular structures. Therefore, in a library with a mixture of a large number of different DNA sequences but with limited copy numbers, it could be challenging to use particular tools to manipulate a few of them. Moreover, random sanitization of DNA molecules has become crucial to ensure data security, potentially giving rise to different research directions [50].

Nonuniform, that is, heterogeneous, efficiency of reactions within a library is also one of the major challenges that scientists are facing. It is not easy to generate a DNA library with an extreme concentration for each sequence. Moreover, molecular handling of these sequences, such as PCR amplification, may magnify the nonuniformity, leading to different efficiencies for further reactions and inaccurate retrieval. Even if the concentration distribution of DNA sequences is uniform, microscopically speaking, the secondary structures will still have slight differences due to the diversity of DNA molecules of different sequences. This will cause different reaction efficiencies and lead to difficulties in information retrieval.

Compared with physical principles such as electrical and optical reactions, which are mostly real-time (femtoseconds or even less), biochemical reactions are usually time consuming (minutes to hours). This is one of the main reasons that DNA molecules are now considered to be the media mostly for cold data storage. Nevertheless, researchers have developed different techniques to increase the speed of writing and reading. By making use of the multi-copy feature of DNA sequences, it is common to conduct massive parallel DNA synthesis and sequencing currently by harnessing mature semiconductor manufacturing techniques. Furthermore, new sequencing techniques such as nanopore sequencing provide the potential for rapid or even real-time information retrieval.

## 9 Future prospects of DNA storage: storage architecture and nanostructure applications

As the demand for scalable, durable, and sustainable data storage solutions grows, DNA storage continues to show immense potential and a promising future. Despite the current challenges, the advancements in storage architecture and integration of nanostructures are revolutionizing the capabilities of DNA storage.

## 10 Storage architecture

DNA storage architecture involves designing systems that efficiently encode, organize, and retrieve data at the molecular level. Future developments in storage architecture should aim to overcome the current limitations of density, retrieval speed, and error correction.

Efficient file labeling is essential for organizing and retrieving data in DNA storage systems. Researchers at North Carolina State University have developed a novel labeling and retrieval method using chemical tags and a nested file address system [51]. This approach significantly enhances the storage capacity and file access capabilities. Similarly, the DNA Enrichment and Nested Separation system improves scalability by advancing labeling and retrieval techniques, making DNA storage systems more adaptable to larger datasets [52].

DNA storage file systems typically follow a three-layer model: the file object layer, file operation layer, and file system interface layer. This hierarchical structure supports efficient file management and random access. File operations, including creation, modification, duplication, and deletion, are facilitated by innovative methods such as PCR-based specific amplification and dehydrated DNA spot shared address systems [53]. These strategies enhance file access efficiency, making DNA storage a more practical and scalable solution for future data storage needs.

It is hoped that the architecture will evolve to tackle challenges related to data retrieval efficiency, which is a key limitation for scalability. A fundamental shift in the architecture is expected with the introduction of high-throughput, parallel DNA synthesis with the capability of isolating every single cluster of a DNA sequence, thus evolving beyond the traditional methods of library synthesis [54], [55]. In this scenario, we will be able to track the information at the stage of writing, which would make it possible to integrate the system with the existing storage architectures. In addition, active data access is crucial for mainstream adoption and will transition DNA storage from passive archival to a dynamic solution, supporting industries such as finance, healthcare, and research. The convergence of bio-inspired architectures and molecular techniques will drive this shift and position DNA as a core element in future data ecosystems.

## 11 Applications of DNA nanostructures

DNA nanostructures enable data encoding and decoding through precise sequence design. For example, DNA strand displacement reactions allow information to be written, erased, and rewritten, making DNA storage systems capable of efficiently handling and storing vast amounts of data. Combining DNA nanotechnology with nanopore sensing has led to the development of rewritable molecular storage systems, such as DNA hard drives, which alter DNA’s 3D structure through controlled molecular attachment and removal to facilitate data writing and erasing [56]. DNA nanostructures also support advanced data encoding methods, such as quaternary encoding using solid-state nanopore platforms, where different-sized DNA multiplex structures increase the storage capacity [57].

Recent developments in nanostructures support a pivotal role for them in DNA-based memory systems. DNA nanostructures can self-assemble to create complex storage systems. By using DNA self-assembly and toehold-mediated strand displacement reactions, researchers have developed multi-bit rewritable DNA storage systems capable of performing logical operations [58]. These systems allow data retrieval through methods such as gel electrophoresis and have potential applications in biomedicine and other fields, including covert product labeling and secure message transmission.

Another critical application of DNA nanostructures in data storage lies in their high density and durability. DNA molecules, with their exceptional physical density and long-term stability, make an ideal medium for storage [59]. The recent advancements include designing specific DNA sequences and nanostructures for various data storage strategies, such as in vivo data storage using CRISPR-Cas systems. These innovations demonstrate the versatility and potential of DNA nanostructures in addressing the growing demand for efficient and durable data storage solutions [59].

In addition, recyclable nanomaterials within nanofluidic systems make DNA storage an eco-friendly and sustainable alternative to conventional systems, which often rely on non-renewable resources [60]. The application of recyclable nanomaterials in nanofluidic systems can make DNA storage an eco-friendly and sustainable alternative [61]. Traditional storage systems often rely on non-renewable resources, whereas nanofluidic technology, combined with the advantages of DNA storage, can significantly enhance data processing efficiency and sustainability through highly integrated and automated platforms [62].

Moreover, innovations such as DNA origami enable the construction of custom-shaped nanostructures (e.g., nanocages), which provide layered and efficient arrangements of DNA molecules. Coupled with SPRI beads, the flexibility of automated purification can be further enhanced [63], [64]. These advancements can facilitate easier data retrieval and long-term stability, which are essential for scaling DNA storage to meet global data demands. Integrating DNA nanostructures with computational systems can also support in-memory computing, offering enhanced processing capabilities and setting the stage for hybrid storage–computation systems.

In conclusion, DNA nanostructures can not only increase the efficiency and accuracy of DNA storage systems but also introduce a novel protocol for how data are stored and manipulated. As these technologies continue to evolve, they are likely to play a central role in achieving scalable, sustainable, and flexible data storage solutions.

## CRediT authorship contribution statement

**Xiaoyuan Xu:** Writing – review & editing, Writing – original draft, Visualization, Conceptualization. **Wen Wang:** Writing – review & editing, Funding acquisition. **Zhi Ping:** Writing – review & editing, Supervision, Funding acquisition, Conceptualization.

## Declaration of Competing Interest

The authors declare that they have no known competing financial interests or personal relationships that could have appeared to influence the work reported in this paper.
